# Surveillance After Pelvic Exenteration: A Systematic Review of Oncological and Functional Follow‐Up

**DOI:** 10.1111/ans.70542

**Published:** 2026-02-23

**Authors:** Adrian Hang Yue Siu, Konrad Joseph, Daniel Steffens, Sharon Carey, Michael Solomon, Cherry Koh

**Affiliations:** ^1^ Surgical Outcomes Research Centre (SOuRCe) Royal Prince Alfred Hospital Camperdown New South Wales Australia; ^2^ Faculty of Medicine and Health, Central Clinical School The University of Sydney Sydney New South Wales Australia; ^3^ Department of Family Practice University of British Columbia British Columbia Canada; ^4^ Department of Colorectal Surgery Royal Prince Alfred Hospital Sydney New South Wales Australia

**Keywords:** colorectal cancer, functional outcomes, oncological follow‐up, pelvic exenteration, post‐operative surveillance, survivorship care

## Abstract

**Background:**

Surveillance following cancer surgery with curative intent serves the purpose of permitting early detection of recurrent disease in addition to monitoring and management of post‐resection functional sequelae. Although recommendations exist for most primary cancers, in the context of radical or re‐operative surgery such as pelvic exenteration (PE), whether these strategies are appropriate is unclear. The purpose of this review was to evaluate the oncological and functional follow‐up strategies after PE.

**Methods:**

A comprehensive literature search was conducted across PubMed, Embase, Medline, and Cochrane databases for studies published between January 2000 and December 2024. Eligible studies reported long‐term oncological and/or functional follow‐up after PE. Studies including other surgical procedures, short‐term (< 12 month) follow‐up, non‐English articles, and case reports were excluded. Data extracted included follow‐up length, frequency, and investigation type. Risk of bias was assessed using the AGREE‐II and ROBINS‐I tools.

**Results:**

Forty‐two studies were included, comprising 39 cohort studies and three non‐original articles. Oncological follow‐up, described in 28 studies, frequently included clinic visits every 3–4 months for the first 2–3 years, transitioning to annual reviews after 5 years. Modalities such as tumour markers, CT and MRI imaging, and colonoscopies varied widely. Functional follow‐up was addressed in 19 studies, utilising validated instruments including the EORTC QLQ‐C30 and SF‐36. Most studies lacked standardisation, and no single article comprehensively addressed both oncological and functional aspects.

**Conclusion:**

Current follow‐up practices after PE exhibit significant heterogeneity, highlighting the need for standardised protocols. Future research should focus on appropriately sized prospective cohort studies and randomised trials to develop evidence‐based guidelines tailored to the unique needs of this high‐risk patient population.

## 1 Introduction

The essential purpose of follow‐up in patients after surgical treatment of their primary cancer is to allow for early detection of local recurrence or systemic disease that may be amenable to further curative intervention. While surveillance practices differ according to geographical location, staging, and clinician preference, a large majority consist of laboratory tests (tumour markers), cross‐sectional imaging (computed tomography [CT] scans), and endoscopic procedures. Despite widespread practice of surveillance protocols, controversy remains over frequency of follow‐up, procedural invasiveness (e.g., risk of perforation in colonoscopies), overall impact on health outcomes, and the substantial economic cost of repeated imaging, endoscopy, and clinic visits, the cost‐effectiveness of which remains unclear, particularly for pelvic exenteration patients [1, 2]. While randomised controlled trials (RCTs) in stages I to III colorectal cancer (CRC) have demonstrated the potential for earlier detection of recurrent disease, improved overall survival has yet to be demonstrated [3, 4].

Beyond oncological surveillance, follow‐up also serves to address long‐term functional consequences of treatment and supporting patients' psychological recovery. This is particularly important in CRC survivors, as psychiatric illnesses such as depression and anxiety are common yet under‐recognised, leading to complications in care, affecting quality of life (QOL), and potentially impacting cancer survival outcomes [5]. Frequent hospital visits can exacerbate the psychological challenges faced by these patients and impact their daily lives, as follow‐up may evoke distress and/or the emotional trauma of their initial diagnosis or surgery [6]. This combination of frequent follow‐up and routine investigations has been colloquially termed as “*scan‐itis*”, defined as the exacerbation of underlying anxiety symptoms and fear of recurrence in routine surveillance imaging of cancer patients [7].

While the appropriateness of surveillance in early‐stage CRC is unclear, many of these studies have focussed on CRC that does not require extended radical resection [8, 9]. For patients with locally advanced or recurrent CRC—pelvic exenteration is offered as a potentially curative surgical modality [10]. This radical procedure, first described by Alexander Brunschwig in 1948 [11], involves en bloc multi‐visceral resection of pelvic organs for curative intent [12]. While pelvic exenteration offers a potentially curative modality for locally advanced malignancies of the central pelvis—most commonly colorectal, urological, or gynaecological—the literature highlights the possibility of treatment failure. A multi‐centre prospective study (*n* = 553) of patients who had undergone surgery for locally recurrent rectal cancer found that a majority of treatment failure was due to local recurrence alone and systemic metastases with or without local recurrence in 14% and 42% of patients, respectively [13]. Therefore, structured post‐operative follow‐up is of utmost importance in this patient group.

In the absence of PE‐specific guidelines, clinicians anecdotally extrapolate follow‐up schedules from their relevant sub‐specialty association—an approach of uncertain validity. For instance, after PE performed by colorectal surgeons for locally advanced or locally recurrent colorectal cancer, follow‐up is often modelled on colorectal specialty guidelines including clinical assessment, tumour markers, imaging with CT and endoscopic surveillance with colonoscopy. While this framework is appropriate for primary colorectal cancers resected with curative intent, its applicability to the locally advanced and recurrent setting is unclear. Moreover, given the extent and complexity of pelvic dissection in PE, the optimal imaging modality and surveillance intervals are unclear, and it remains further uncertain whether CT alone constitutes sufficient sensitivity for detecting early or subtle pelvic recurrence. As PE is performed not only for locally advanced or recurrent colorectal cancer but also for locally advanced gynaecological and urological malignancies, the extent of resection provides a unique opportunity to capture broader perspectives on post‐operative surveillance, irrespective of tumour origin. By considering different follow‐up strategies across tumour types that share this complex surgical approach, this study aimed to evaluate oncological and functional follow‐up practices after pelvic exenteration surgery from a procedure‐specific perspective.

## 2 Methods

The review followed Cochrane [14] and PRISMA [15] methodological guidelines for systematic reviews. The review protocol was prospectively registered with the International Prospective Register of Systematic Reviews (PROSPERO ID CRD42024501031).

### 2.1 Search Strategy

An electronic search for relevant publications was performed across the following databases: PubMed, Embase, Medline, and the Cochrane Collaboration Database. The search focused on primary research articles, including RCTs and observational cohort studies, examining follow‐up after pelvic exenteration from 1 January 2000 to 31 December 2024. Meta‐analyses, systematic reviews, editorials, and expert review articles, consensus guidelines were included but reported separately from the original research articles.

The search strategy was developed and refined with the assistance of a medical librarian. The following keywords were used to perform the search: “pelvic exenteration”, “follow up”, “recurrence assessment” and “surveillance”. All relevant Medical Subject Headings (MeSH) terms and accompanying search strategies are included in Appendix S1 for reference. Results from each database were collected and imported into reference management software (Endnote 20) [16]. Duplicates were removed before title and abstract screening. A pre‐determined selection criteria were applied for full text analysis. Studies that did not meet the inclusion criteria were excluded. Additionally, the reference lists of identified articles were manually screened for potentially relevant studies.

### 2.2 Nomenclature of Pelvic Exenteration

Within the literature, there is currently no universally accepted nomenclature for pelvic exenteration surgery, where terms such as “extended”, “extended radical” or “anterior/posterior/total pelvic exenteration” are used variably in the literature. Therefore, for the purposes of this systematic review, a pragmatic definition was adopted, where “pelvic exenteration” was defined as any en bloc multi‐visceral pelvic resection extending beyond total meso‐rectal excision.

### 2.3 Selection Process (Inclusion and Exclusion Criteria)

Title and abstract screening, and full‐text review were conducted by two independent authors (AS, KJ). Disagreements were resolved upon consensus or with mediation by a third reviewer (DS). Articles were retrieved for full text analysis if they included the following pre‐determined selection criteria: (a) reported on patients who underwent pelvic exenteration for either colorectal, urological or gynaecological malignancy; (b) reported on the long‐term follow‐up of pelvic exenteration (≥ 12 months); (c) reported on oncological or functional outcomes of follow‐up.

Studies were excluded from analysis if: (a) they included other surgical procedures; (b) reported on only short‐term follow up after pelvic exenteration; (c) did not report on the outcome of interest or surgical technique of interest; (d) case reports; (e) conference abstracts; (f) non‐English studies; (g) non‐human population; (h) duplication of data from the same unit or hospital (the most relevant article was retained).

### 2.4 Data Collection Process

Two independent authors (AS, KJ) extracted the following data variables into a data extraction form: title and reference details (first author, journal, year, country), patient demographics (number of patients in study, gender, and age), type of study, malignancy characteristics (colorectal, urological, gynaecological), methodology of follow‐up, and follow‐up data outcomes (oncological or functional). All data were recorded independently by both reviewers in separate data extraction forms and were compared at the end extraction to limit selection bias. Duplication of data was removed, and any disparities were clarified through consensus or via mediation by a third reviewer (DS) and further review of the literature.

### 2.5 Data Items (Outcomes of Interest)

The following outcomes were extracted from the included studies: oncological follow‐up (length, frequency of investigations and type of investigations); and functional follow‐up (length of follow‐up, frequency, and methodology) for bowel function, urinary function, sexual function, lower limb function and pain.

### 2.6 Risk of Bias Assessment

Risk of bias for included primary research studies was performed by two authors (AS, KJ) independently, using The Cochrane tool Risk of Bias in Non‐randomised Studies of Interventions (ROBINS‐I V2) for observational studies, and Risk of Bias 2 (RoB2) for randomised studies [17, 18]. Studies were categorised as having a ‘lower’ risk of bias when assessed as ‘low’ or ‘moderate’ using ROBINS‐I V2, or ‘low’ using RoB2. Conversely, studies assessed as ‘serious’ or ‘critical’ using ROBINS‐I V2, or ‘unclear’ or ‘high’ using RoB2, were identified as having a ‘higher’ risk of bias. Guideline appraisal was performed using The Appraisal of Guidelines for Research & Evaluation (AGREE) II instrument [19]. Each guideline was assigned an overall quality rating on a scale of 1 to 7, where ‘1’ represents the lowest quality, ‘2’ is low quality, ‘3’ is low‐to‐moderate quality, ‘4’ is moderate quality, ‘5’ is moderate‐to‐high quality, ‘6’ is high quality and ‘7’ is the highest quality. Additionally, each guideline was recommended for use as ‘Yes’, ‘Yes with modifications’ or ‘No’.

## 3 Results

### 3.1 Literature Search

**FIGURE 1:**
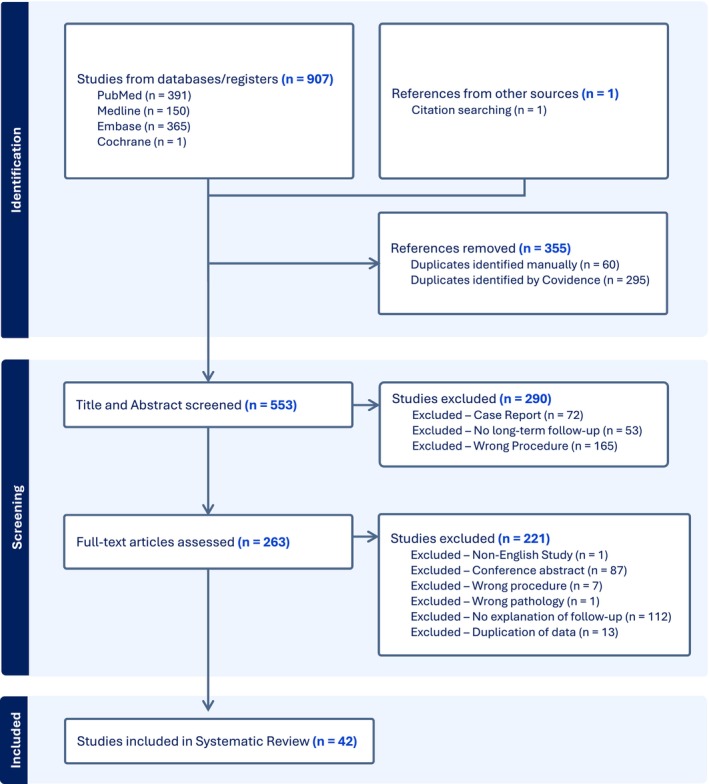
PRISMA flow diagram for this systematic review, showing selection of articles for review and reasons for exclusion. PRISMA = Preferred Reporting Items for Systematic Review and Meta‐Analyses.

Following a comprehensive literature search, there were 553 references identified after removal of duplicates. After title and abstract screening, 290 articles were excluded, and 263 articles were retrieved for full‐text analysis. The exclusion and inclusion criteria were applied to the retrieved articles, and 42 articles were deemed eligible for inclusion. Full details of the included and excluded articles are illustrated in the PRISMA flow diagram [20] (Figure 1).

### 3.2 Overall Study Characteristics

A total of 42 articles were included in the systematic review, 39 cohort studies, 2 consensus guidelines, and 1 expert review article. There were no RCTs eligible for analysis. Of the 42 articles, 23 focused on oncological follow‐up, 11 on functional follow‐up, and 8 covered both oncological and functional aspects. When broken down by oncology type, 15 articles focused solely on colorectal cancer, 15 on gynaecological cancer, and 12 evaluated a mixture of colorectal, gynaecological and urological malignancies.

Of the 39 cohort studies, 10 studies utilised prospective data collection, and 29 were retrospective. Of the cohort studies, 25 were categorised as ‘lower’ risk of bias, and 14 were categorised as ‘higher’ risk. The 2 consensus guidelines were assessed as being of ‘moderate‐to‐high quality’ [21] and ‘high quality’ [22], with both receiving a recommendation for use of ‘yes, with modifications’. The final non‐original article, an expert review, was not appraised due to its methodology and the absence of validated tools for evaluating this type of publication.

### 3.3 Oncological Follow‐Up

A total of 28 cohort studies were included for oncological follow‐up, of which 21 had a ‘low risk of bias’. The total number of patients with oncological follow‐up was 2391, and sample sizes (*n*) for these included articles ranged from 6 to 388 patients. For the frequency of clinical follow‐up, a majority of articles (21 studies) endorsed implementing a 3–4 month review interval after surgery for at least the first 2–3 years, before transitioning to biannual consultations until the 5th year post‐operatively, and annually thereafter [23, 24, 25, 26, 27, 28, 29, 30, 31, 32, 33, 34, 35, 36, 37, 38, 39, 40, 41, 42, 43]. Two of the studies reported a higher frequency of follow‐up in the first year after surgery, reviewing their patients monthly for the first 6 months [44, 45], before transitioning to biannual and less frequent follow‐up. One study reported less frequent follow‐up from the outset, where patients were reviewed at 6‐weeks, 6‐months and then annually [46]. There were four studies that did not specify the frequency of follow‐up regime [47, 48, 49, 50], and three of these studies were categorised as ‘high risk of bias’. As part of follow‐up consultations, 10 of the studies [23, 24, 25, 30, 32, 35, 39, 41, 42, 46] reported performing physical examinations and monitoring of laboratory tests including tumour markers (CEA, CA 125, CA 19‐9).

There was significant variation in the frequency and modalities of imaging techniques used for follow‐up in the included articles. Fourteen of the studies reported using thoraco‐abdominopelvic CT with or without positron emission tomography (PET) at varying stages of follow‐up either biannually or annually for 5–10 years post‐operatively [23, 24, 31, 32, 33, 34, 37, 38, 40, 41, 42, 43, 45, 50]. Six of the studies reported using transabdominal and pelvic ultrasound (US) for follow‐up in addition to CT [26, 29, 31, 36, 39, 50], however, five of these studies focused on follow‐up for gynaecological cancer. Four studies reported the use of magnetic resonance imaging (MRI), where this imaging modality was used in addition to or in place of CT [23, 33, 39, 49]. However, only one study reported different frequencies of CT and MRI based on the extent of resection, where an additional baseline MRI was performed at 3 or 6 months when patients had undergone an R1 resection [49].

The final modality for follow‐up was colonoscopy to assess for recurrence, which was reported in seven articles. Four of these articles reported colonoscopies at 1 year post‐operatively, with increasing intervals of 3–5 years [32, 34, 35, 38], while two of the articles reported colonoscopies annually for the first 5 years [40, 50]. One further article reported the use of colonoscopy at 1 year after surgery, but highlighted that further investigation was indicated by clinical findings [23]. On review of the oncology types, five studies were colorectal cancer, and two studies were colorectal and gynaecological malignancy. An overview of all primary articles for oncological follow‐up, study design, patient population, risk of bias, and follow‐up methods can be found in Table 1.

**TABLE 1:** Overview of primary research articles for oncological follow‐up (*n* = 28).

References	Study design	Patients (male, female, median age)	Oncology	Follow‐up methodology	Risk of bias
Ali Uraiqat et al. [23]	Retrospective cohort	30 (8, 22, 55 years)	Colorectal and gynaecological	Patients attended follow‐ups every 3 months initially, extending to 6‐month intervals after 2 years. At each visit, a physical exam including DRE was performed, and carcinoembryonic antigen (CEA) measured. Pelvic MRI and CT of the chest, abdomen, and pelvis were performed every 12 months. Colonoscopy at 1 year after surgery, then as indicated.	Lower
Chew et al. [24]	Retrospective cohort	25 (16, 9, 62 years)	Colorectal and gynaecological	Follow‐up consisted of 3‐monthly clinic visits for at least 2 years. Follow‐up included monitoring of CEA level (each visit) and total‐body CT (once annually for the first 2 years).	Lower
Chiva et al. [44]^a^	Retrospective cohort	6 (0, 6, 47 years)	Gynaecological	Patients were followed monthly for the first 6 months, every 3 months for the remainder of the first year, every 4 months for the second year, and then bi‐annually until 5 years.	Higher
Chokshi et al. [25]	Retrospective cohort	54 (21, 32, 56 years)	Colorectal, gynaecological, and urological	Patients were given a follow‐up clinic appointment in 2 weeks. They were followed every 3 months for the next year and every 6 months after the first year. CEA levels were drawn at each visit; any increase in CEA or symptomatology prompted repeat imaging—CT/PET.	Lower
Cortinovis et al. [26]^a^	Retrospective cohort	16 (0, 16, 56 years)	Colorectal and gynaecological	Evaluations were scheduled every 4–6 months for the first 2–5 years after surgery, and yearly thereafter. Follow‐up evaluations consisted of pelvic examination and transabdominal US scan/CT.	Higher
Espinosa‐de‐Los‐Monteros et al. [45]	Retrospective cohort	10 (3, 7, 53 years)	Colorectal	Follow‐up was performed weekly for the first month, once a month for the following 5 months, and once every 6 months thereafter. Follow‐up included physical examination and review of CT scans.	Lower
Fleisch et al. [47]^a^	Retrospective cohort	203 (0, 203, 56 years)	Gynaecological	Follow‐up was obtained in a combination of patient records, telephone interviews with patients/relatives/primary care physicians/specialists. Data were collected using a standardised follow‐up questionnaire covering local or distant recurrences, and death.	Higher
Goldberg et al. [27]^a^	Retrospective cohort	103 (0, 103, 53 years)	Colorectal and gynaecological	Patients were followed in the office every 3 months for the first 2 years, every 6 months until the fifth year and annually thereafter. Follow‐up included evaluation of the renal tract via CT imaging.	Lower
Gould et al. [46]	Retrospective cohort	388 (242, 146, 59 years)	Colorectal	Follow‐up was offered at 6 weeks and 6 months, then annually for five years. Follow‐up included imaging and serial CEA measurements as per NICE guidance.	Lower
Guimaraes et al. [28]	Retrospective cohort	30 (4, 26, 52 years)	Colorectal and gynaecological	Patients were followed up with physical exams, CT scan, Chest X‐ray, chemistry profile, and CBC at a 3‐month interval for the first 2 years, at a 6‐month interval from 3 to 5 years, and annually thereafter.	Lower
Hockel [29]	Prospective cohort	74 (0, 74 52 years)	Gynaecological	Patients were followed up with an interview and physical examination, supplemented by pelvic and abdominal ultrasound investigation and laboratory testing. Follow‐up visits were scheduled in 3‐month intervals during the first 2 years after treatment, and biannually thereafter.	Lower
Jurado et al. [30]	Retrospective cohort	48 (0, 48, 50 years)	Gynaecological	Follow‐up evaluations included physical and pelvic exam, CBC, chemistry profiles, tumour markers, chest X‐rays and total body CT scan, scheduled at 3–4‐month intervals.	Lower
Kanao et al. [31]	Prospective cohort	28 (0, 28, 54 years)	Gynaecological	Follow‐up consisted of pelvic examination, US, and blood tests at 1‐ to 3‐month intervals and CT at 3‐ to 6‐month intervals.	Lower
Kazi et al. [32]	Retrospective cohort	288 (155, 130, 45 years)	Colorectal	Patients were followed up with a total‐body CT scan at 3 and 6 months, then 6‐monthly until 3 years, and annually from 3 to 5 years. A physical exam was performed, and CEA was sampled at every visit. Colonoscopy was indicated at 1 and 3, and every 5 years thereafter.	Lower
Khan et al. [48]^a^	Retrospective cohort	104 (75, 29, 61 years)	Colorectal	Patients were reviewed by the colorectal team with telephone and face‐to‐face follow‐up. Once satisfactory progress was made, they were often referred back to their local region for further oncological follow‐up.	Lower
Kulu et al. [33]	Retrospective cohort	187 (79, 104, 59 years)	Colorectal, gynaecological, and urological	Clinical follow‐up included a physical examination every 3 months for 2 years and then annually until 5 years. A CT or MRI of the abdomen and pelvis was performed 3 months after surgery and then every 6–12 months, according to clinical circumstances.	Lower
Kumar et al. [34]	Retrospective cohort	131 (57, 43, 45 years)	Colorectal	Follow‐up was arranged every 4 months for the first 2 years, every 6 months for the next 3 years, and annually thereafter. Evaluations included clinical assessment, and serum CEA. Total body CT was performed yearly. Colonoscopy was performed at 1, 3, and 5 years.	Lower
Li et al. [35]	Retrospective cohort	59 (29, 30, 54 years)	Colorectal and gynaecological	Patients were followed up every 3 months for the first 3 years postoperatively, every 6 months for 4–5 years postoperatively, and annually after 5 years. Routine follow‐up included whole‐body CT and tumour markers. Rectal cancer patients underwent colonoscopy in the 1st, 3rd, and 5th postoperative years.	Lower
Puntambekar et al. [36]	Retrospective cohort	74 (0, 74, 50 years)	Gynaecological	Patients were followed up once a month for 3 months and then once every 3 months for the first 2 years, every 6 months for the next 3 years, and annually for the following 5 years. At every follow‐up, urine analysis was performed. US of the abdomen and pelvis and a PET scan was performed every 6 months to determine the disease‐free interval.	Lower
Quyn et al. [49]	Prospective cohort	35 (16, 18, 57 years)	Colorectal	Patients were followed up by clinical review and CT scanning for 5 years post‐operatively. The first institution performed MRI at 6 and 12 months and CT at 12 and 24 months. Following these time points, the follow‐up was guided by clinical oncology. If a patient had an R1 resection, an additional baseline MRI was performed at 3 months. In the second institution, exenterations were followed up at 6 and 12 months with CT and then annual CT surveillance. An additional MRI was performed at 3 or 6 months for cases with a R1 resection.	Higher
Sando et al. [37]	Retrospective cohort	40 (29, 11, 61 years)	Colorectal and gynaecological	Patients were followed up with blood tests every 3 months for the first 3 years and then every 6 months up to 10 years. CT was performed every 6 months for 10 years.	Lower
Sanfilippo et al. [38]	Retrospective cohort	45 (26, 19, 55 years)	Colorectal	Patients were followed up every 3–4 months for 2 years, every 6 months up to 5 years, and yearly thereafter. A clinical examination and proctoscopy, abdominopelvic CT and chest X‐ray was obtained every 6 months for 2 years and yearly thereafter. Colonoscopy was performed at intervals of 1–3 years depending on the findings of the prior study.	Lower
Stanca et al. [39]^a^	Retrospective cohort	47 (0, 47, 54 years)	Gynaecological	Patients were followed up quarterly for the first 2 years, biannually for the next 3 years, and then annually. Before each visit, laboratory examinations and an annual total body CT or MRI were performed. During the visits, local and general clinical examinations, pap‐smear of the vaginal vault, and ultrasound examinations were performed.	Higher
Uehara et al. [43]^a^	Retrospective cohort	35 (27, 8, 66 years)	Colorectal	Patients were followed at 3‐month intervals up to 5 years. Recurrence was diagnosed based on CT, which were performed at 6‐month intervals for up to 5 years post‐operatively.	Lower
Visokai et al. [50]	Retrospective cohort	28 (20, 8, 68 years)	Colorectal	For follow‐up, transabdominal US was performed, and tumour markers were checked biannually. Abdominal CT scan and colonoscopy were performed annually. Chest X‐ray was performed every 2 years.	Higher
Vitelli et al. [40]	Retrospective cohort	26 (12, 14, 62 years)	Colorectal	Follow‐up was performed regularly every 3 months for the first 3 years, then every 6 months until the fifth year, and annually thereafter. Total‐body CT and colonoscopy was performed every year for the first 5 years.	Lower
Yang et al. [41]	Retrospective cohort	40 (32, 8, 59 years)	Colorectal	Patients attended standardised follow‐up every 3 or 6 months. At each visit a physical examination DRE was performed, and CBC, liver function tests, and CEA were drawn. Total‐body CT with or without PET‐CT, were performed every 6 months.	Lower
Zoucas et al. [42]^a^	Retrospective cohort	85 (68, 17, 62 years)	Colorectal, gynaecological, and urological	Patients were followed every 3 months with a serum CEA. All underwent total‐body CT with and without PET every 6 and 12 months respectively.	Higher

Abbreviations: CA125, cancer antigen 125; CA19‐9, cancer antigen 19‐9; CEA, carcinoembryonic antigen; CBC; complete blood count, CT; computed tomography; DRE; digital rectal examination, MRI, magnetic resonance imaging; NICE, National Institute for Health and Care Excellence; PET, Positron emission tomography; SCCA, squamous cell carcinoma antigen; US, ultrasound.

^a^ Study included both oncological and functional follow‐up.

There were three non‐original research articles which were considered lower level evidence but included in this systematic review due to their particular focus on pelvic exenteration oncological follow‐up. The expert review article [51] emphasised the importance of CT or MRI in assessing recurrence, and suggested that MRI may be superior to CT in detecting recurrent disease after pelvic exenteration. The two other articles were consensus guidelines [21, 22], and both studies emphasised the deficiency of literature and absence of specific follow‐up protocols following pelvic exenteration. One consensus guideline [21] recommended clinic follow‐up at 3‐month intervals for the first 2 years, before transitioning to biannual intervals until 5 years post‐surgery, a schedule consistent with many cohort studies. Similarly, the other consensus guideline [22] focused on follow‐up on a regular basis, with decreasing frequency based on clinical need over time. Additionally, this article reported on the importance of annual thoraco‐abdominopelvic CT, colonoscopy in 3‐year intervals if no new lesion is found, and appropriate follow‐up by other specialties when extended procedures were performed in the initial operation. An overview of all non‐original articles for oncological follow‐up, study design, and main follow‐up findings can be found in Table 2.

**TABLE 2:** Overview of non‐original articles for oncological follow‐up (*n* = 3).

References	Study design	Oncology	Main follow‐up findings	Appraisal
Lakhman et al. [51]	Review article	Gynaecological	The normal imaging appearance after PE is highly variable secondary to the variations in the scope of the pelvic resection and types of reconstructive procedures. New asymmetric soft tissue or enhancement at CT scans or MR imaging should be regarded with suspicion. MR imaging may be better than CT for detection of recurrent disease due to its superior soft‐tissue contrast. FDG PET/CT may also help identify disease recurrence by demonstrating hypermetabolic tumour or lymphadenopathy.	N/a
Laporte et al. [21]	Consensus guidelines	Gynaecological	There are no studies addressing follow‐up regimens, specifically after PE. Follow‐up recommendations are similar among the European Society of Gynaecological Oncology, the National Comprehensive Cancer Network, and the International Federation of Gynaecology and Obstetrics. The follow‐up objectives for patients who undergo PE with curative intent are the detection of new recurrence, the identification and management of treatment‐related complications, and the provision of psychosocial and educational support to restore quality of life. *Recommendation*: After the procedure, history and physical examination should be considered at 3‐month intervals for the first 2 years and at 6‐month intervals from 2 to 5 years. Laboratory and imaging tests should be individualised and ordered according to the clinical picture.	*Overall quality*—5 (moderate‐to‐high quality) *Recommendation for use*—yes, with modifications
PelvEx Collaborative [22]	Consensus guidelines	Colorectal	Recommendation: Patients should remain under structured medical review for at least 5 years after pelvic exenteration. While no universally accepted follow‐up schedule exists for this operation, surveillance may broadly reflect standard colorectal cancer protocols, adapted to individual risk and recovery. Clinical assessment—including physical examination, review of symptoms and wellbeing, and relevant blood tests—should occur regularly, with visits spaced further apart over time according to clinical stability. There is no clear evidence supporting more intensive early review for complex reconstructions; therefore, annual assessment is generally sufficient, with a final comprehensive review at 5 years. Routine surveillance should include annual total‐body CT imaging, accompanied by tumour‐marker testing such as CEA where appropriate. Additional laboratory studies are guided by organ involvement. PET‐CT may clarify metastatic disease or distinguish recurrent tumour from postoperative changes but has limited use in mucinous adenocarcinoma. Colonoscopy remains indicated lifelong when the index tumour was colorectal in origin—typically at three‐year intervals, adjusted by the endoscopist based on prior findings or incomplete examinations. Following extended resections (e.g., involving bone), coordinated rehabilitation is essential, as functional recovery may take several months to a year. Collaboration with orthopaedic and rehabilitation teams should form part of ongoing care. After urinary diversion, long‐term follow‐up should include periodic renal function testing and annual imaging of the upper tracts, ideally under the oversight of the original surgeon with input from nephrology and other MDT members as clinically indicated.	*Overall quality*—6 (high quality) *Recommendation for use*—yes, with modifications

Abbreviations: CT, computed tomography; FDG, fluorodeoxyglucose; MDT, multidisciplinary team; MR, magnetic resonance; PE, pelvic exenteration; PET, Positron emission tomography.

### 3.4 Functional Follow‐Up

In total, functional follow‐up scores were measured from 1402 patients across 19 studies. Sample sizes (*n*) for these articles ranged from 6 to 287 patients. A slight majority, 11 studies were classified as “high risk of bias.” For functional follow‐up, a large variety of validated instruments and survey techniques at various timepoints were used post‐operatively. The most commonly used instrument in seven studies [39, 42, 52, 53, 54, 55, 56] was the colorectal cancer European Organisation for Research and Treatment of Cancer (EORTC) Quality of Life Questionnaire (QLQ) C30. One of these studies [55], additionally used other validated instruments for fatigue (Brief Fatigue Inventory [BFI]), pain (Brief Pain Inventory—Short Form [BPI‐SF]), and depression (Centre of Epidemiologic Studies Depression Scale [CES‐D]). The next most commonly used instrument in 3 studies [26, 56, 57] was the Short‐Form 36 (SF‐36) for health‐related QOL, followed closely by the Functional Assessment of Cancer Therapy—Colorectal (FACT‐C), which was used in two studies [57, 58]. For sexual function, this was explored in one study [26] through the Female Sexual Function Index questionnaire. Urinary function was assessed in a number of different ways across six different studies [27, 44, 48, 59, 60, 61] from questionnaires—Urogenital Distress Inventory (UDI‐6), laboratory tests (blood urea nitrogen, creatinine levels, serum electrolytes), CT and/or urogram, and interviews in follow‐up clinics. Only one study [59] assessed bowel function using the validated Faecal Incontinence Severity Index (FISI), and there were two studies [43, 47] where follow‐up was obtained through patient records and interviews with patients about their functional symptoms. An overview of all primary articles for functional follow‐up, study design, patient population, risk of bias, and main follow‐up methods can be found in Table 3.

**TABLE 3:** Overview of primary research articles for functional follow‐up (*n* = 19).

References	Study design	Patients (male, female, median age)	Oncology	Main follow‐up methodology	Risk of bias
Angeles et al. [52]	Prospective cohort	56 (0, 56, 50 years)	Gynaecological	QOL was assessed using the EORTC QLQ‐C30 (version 3.0) and the EORTC QLQ‐OV28 questionnaires before, and at 1, 3, 6, and 12 months post‐operatively.	Lower
Arnaboldi et al. [62]	Prospective cohort	49 (0, 49, 54 years)	Gynaecological	The psychological distress management protocol includes the administration of the Psychological Distress Inventory (PDI) and the Mini‐mental Adjustment to Cancer (Mini‐MAC) scale. These were performed before surgery and annually thereafter.	Higher
Berretta et al. [59]	Retrospective cohort	22 (0, 22, 65 years)	Gynaecological	To evaluate residual bladder and rectal function at 6‐months post‐surgery, all patients were asked to complete the validated short version of the Urogenital Distress Inventory (UDI‐6) questionnaire to detect any urinary disorders, and the validated Faecal Incontinence Severity Index (FISI) questionnaire for detection of faecal incontinence.	Lower
Chiva et al. [44]^a^	Retrospective cohort	6 (0, 6, 47 years)	Gynaecological	During routine follow‐up, patients were checked for urinary symptoms including urinary leakage, grade of continence, bleeding, infections, lumbar pain, and episodes of overflow incontinence. BUN and creatinine levels were checked every 3 months to evaluate renal function.	Lower
Cortinovis et al. [26]^a^	Retrospective cohort	16 (0, 16, 56 years)	Colorectal and Gynaecological	Patients were administered 2 questionnaires at baseline and at 12‐month follow‐up. The first questionnaire was the SF‐36 to measure health‐related QOL. Sexually active patients were administered the second questionnaire, the Female Sexual Function Index (Italian version).	Higher
Fleisch et al. [47]^a^	Retrospective cohort	203 (0, 203, 55 years)	Gynaecological	Follow‐up was obtained in a combination of accessible patient records, telephone interviews with patients/relatives/primary care physicians/specialists. Data were collected using a standardised questionnaire covering postoperative complications, re‐admissions, reinterventions or re‐surgery, and QOL aspects.	Higher
Goldberg et al. [27]^a^	Retrospective cohort	103 (0, 103, 53 years)	Colorectal and Gynaecological	Surviving patients were surveyed regarding their satisfaction with the vaginal reconstruction and their sexual function after the surgery. Patients with continent urinary diversion were also questioned regarding their satisfaction with the procedure and related issues.	Lower
Khan et al. [48]^a^	Retrospective cohort	104 (75, 29, 61 years)	Colorectal	Urology follow‐up was typically at 3 months with patient review, using CT and/or loop‐o‐gram studies for conduits to ensure free reflux through the uretero‐enteric anastomosis.	Lower
Liedl et al. [60]	Retrospective cohort	16 (0, 16, 51 years)	Gynaecological	Follow‐up was either through outpatient review or scheduled follow‐up operation (e.g., bladder neck suspension). The main long‐term follow‐up were postoperative continence grade, urinary tract infections, micturition problems, residual urine, ureteric reflux, as well as urinary tract‐related postoperative complications or effects on general condition (QOL).	Lower
Lin et al. [58]	Retrospective cohort	122 (35, 87, 56 years)	Colorectal	Patients completed a series of questionnaires pre‐operatively (baseline) as well as at 1, 3, 6, 9, and 12 months after PE. The questionnaires were PROCTCAE and the FACT‐C.	Higher
Martinez et al. [53]	Prospective cohort	61 (0, 61, 60 years)	Gynaecological	QOL was assessed using the EORTC QLQ‐C30 (version 3.0) and the EORTC QLQ‐OV28 questionnaires before surgery, and then 1, 3, 6, and 12 months after the procedure.	Higher
Radwan et al. [54]	Retrospective cohort	56 (19, 37, 64 years)	Colorectal	Patients were asked to complete the EORTC QLQ‐C30 questionnaire at baseline (preadmission appointment) and 2 weeks after surgery. Subsequent questionnaires were requested at 3‐, 6‐, 12‐ and 24‐months following surgery.	Higher
Rezk et al. [55]	Prospective cohort	16 (0, 16, 58 years)	Gynaecological	Patients were interviewed pre‐operatively, and at 3, 6, and 12 months after PE for physical/psychological symptoms. The study consisted of the EORTC QLQ‐C30 (version 3.0) and its colorectal cancer (EORTC QLQ‐CR38) and muscle invasive bladder cancer (EORTC QLQ‐BLM30) modules, the Brief Fatigue Inventory (BFI), the Brief Pain Inventory‐Short Form (BPI‐SF), the Instrumental Activities of Daily Living (IADL), the Centre for Epidemiologic Studies Depression Scale (CES‐D), and the Impact of Events Scale‐Revised (IES‐R).	Higher
Stanca et al. [39]^a^	Retrospective cohort	47 (0, 47, 54 years)	Gynaecological	Patients were followed‐up with translated QOL questionnaires issued from the EORTC QLQ‐C30 and EORTC QLQCX24. These questionnaires were performed at one timepoint (average 26‐months) post‐operatively.	Higher
Steffen et al. [57]	Prospective cohort	287 (176, 111, 60 years)	Colorectal	SF‐36 and the Functional Assessment of Cancer Therapy – Colorectal (FACT‐C) instruments at baseline (before surgery) and at 6, 12, 18, 24, 30, 36, 48 and 60 months after PE surgery.	Lower
Sukumar et al. [61]	Retrospective cohort	12 (8, 4, 54 years)	Colorectal and urological	Follow‐up protocol included 3‐monthly serum electrolyte evaluations, US of upper tracts (Uro‐grams), and annual CT scans of the abdomen and pelvis.	Higher
Uehara et al. [43]^a^	Retrospective cohort	35 (27, 8, 66 years)	Colorectal	Follow‐up included recording whether patients had difficulty in ambulation post‐operatively.	Lower
Zanatto et al. [56]	Retrospective cohort	106 (52, 54, 61 years)	Colorectal, gynaecological, and urological	Two QOL questionnaires were used, the SF‐36 version 2, and the EORTC QLQ‐C30 version 3.0 in Portuguese.	Higher
Zoucas et al. [42]^a^	Retrospective	85 (68, 17, 62 years)	Colorectal, gynaecological, and urological	All patients who underwent surgery after May 2005 were asked to complete a questionnaire post‐operatively, and follow‐up of these patients is ongoing annually. The questionnaire used was the EORTC QLQ‐C30 and was performed at 4 months and 16 months post‐operatively.	Higher

Abbreviations: CT, computed tomography; EORTC, European Organisation for Research and Treatment of Cancer; PE, pelvic exenteration; PROCTCAE, Patient‐Reported Outcomes version of the Common Terminology Criteria for Adverse Events; QLQ‐BLM30, Quality of Life Questionnaire—Muscle Invasive Bladder Cancer Module; QLQ‐CR38, Quality of Life Questionnaire—Colorectal Cancer Module; QLQ‐CX24, Quality of Life Questionnaire—Cervical Cancer Module; QLQ‐C30, Quality of Life Questionnaire—Core Questionnaire; QLQ‐OV28, Quality of Life Questionnaire—Ovarian Cancer Module; QOL, quality of life; SF‐36, Short‐Form 36; US, ultrasound.

^a^ Study included both oncological and functional follow‐up.

## 4 Discussion

The essential purpose of follow‐up after surgical treatment is to allow for early detection of local recurrence or systemic disease that may be amenable for further curative management, and to afford the opportunity to address functional and psychological challenges, especially after major surgery [63]. This systematic review provides an overview of the literature surrounding oncological and functional follow‐up approaches after pelvic exenteration. In this review, there were 20 and 11 studies that focused on oncological and functional follow‐up respectively, and 8 studies which included elements of both. From these 39 cohort studies, 25 were categorised as ‘lower’ risk of bias, and their follow‐up is detailed in the methods section. Almost all of the studies were single‐institution series, where follow‐up protocols varied in intensity, frequency and/or modality. Furthermore, the absence of RCTs has prevented direct comparisons of follow‐up protocols to evaluate for differences in recurrence rates, post‐operative outcomes, or patient satisfaction. This holds significant implications for patients who have undergone pelvic exenteration, as the high morbidity associated with surgery necessitates a careful balance between close follow‐up and avoiding over‐investigation, which can cause iatrogenic harm and adversely affect long‐term QOL [5, 6, 7].

As suggested in a majority of the studies (21 articles) for oncological follow‐up, patients were reviewed every 3–4 months for the first 2–3 years post‐operatively, before transitioning to biannual consultations until the 5th year, and then annually [23, 24, 25, 26, 27, 28, 29, 30, 31, 32, 33, 34, 35, 36, 37, 38, 39, 40, 41, 42, 43]. This follow‐up schedule which focuses on early surveillance, broadly aligns with the period of highest recurrence risk, with median time to recurrence after pelvic exenteration for colorectal, gynaecological, and urological malignancies ranging from 6 to 24 months [64, 65, 66]. Follow‐up consultations include physical examination, blood sampling, imaging, and monitoring of tumour markers (e.g., CEA) every 3–6 months, and adheres to previously established follow‐up recommendations for early‐stage colorectal cancer [67]. However, applying these recommendations for patients who do not require extended radical resection to pelvic exenteration patients remains unsubstantiated in the literature. In addition to conventional tumour markers, emerging biomarkers such as circulating tumour DNA (ctDNA) have shown promise in colorectal cancer surveillance. While not utilised in any of the studies included in this review, ctDNA is currently being investigated in ongoing trials, and may represent a valuable adjunct in this high‐risk population in the future [68]. However, this is a constantly evolving field, and future studies are required to investigate its role in advanced disease settings. Difference in follow‐up modalities were also observed based on oncology type, where gynaecological cancer studies [26, 29, 31, 36, 39, 50] favoured transabdominal and pelvic US. This modality was used in addition to biannual or annual thoraco‐abdominopelvic CT with or without PET, which formed the mainstay for assessment of oncological recurrence regardless of oncology type [23, 24, 31, 32, 33, 34, 37, 38, 40, 41, 42, 43, 45, 50].

A limited number of studies (four articles) incorporated MRI for assessment of oncological recurrence, either in combination with or as an alternative to thoraco‐abdominopelvic CT [23, 33, 39, 49]. The frequency of MRI use varied, ranging from every 3–6 months to annually. One study by Quyn et al. [49] further described two different follow‐up strategies with minor differences and performed an additional baseline MRI at 3 or 6 months for cases with R1 resection. Although this study uniquely presented two follow‐up approaches, there was no comparison for recurrence or survival outcomes. While MRI is considered superior to CT for detecting recurrent pelvic disease due to its enhanced soft‐tissue contrast [51], its routine use for surveillance is limited by higher costs, contraindications (especially in patients with metal implants), patient discomfort, and its limited ability to detect systematic recurrences such as lung, para‐aortic, or liver metastases [69]. Alternatively, the use of PET and/or fluorodeoxyglucose PET (FDG‐PET) was described in several studies [23, 35, 50] as an alternative to CT for evaluation of local recurrence, metastatic disease, or during cases of diagnostic uncertainty. Therefore, although multiple imaging modalities are available for assessing recurrence, there is no definitive recommendation regarding optimal modality or frequency of follow‐up.

Another modality for follow‐up was colonoscopy, which was primarily reported in seven articles that included colorectal oncology [23, 32, 34, 35, 38, 40, 50]. Four studies reported performing colonoscopy at 1, 3 and 5 years post‐operatively [32, 34, 35, 38], aligning with previously established colorectal cancer guidelines [70]. However, two studies reported performing annual colonoscopies for 5 years [40, 50]. Both of these follow‐up strategies differed from the recommendations of the PelvEx Collaborative consensus guidelines [22], which suggested colonoscopies at intervals of 3 years if no new lesions were detected. While colonoscopy remains an integral investigation for colorectal cancer surveillance [71], it carries additional risks, including those associated with anaesthesia, perforation, and bleeding, compared to non‐invasive imaging modalities [72]. This disparity in frequency of invasive investigations holds significant implications for this patient population, where such procedures should be minimised, especially following a major multi‐visceral operative procedure with significant risk of morbidity and mortality [73].

Two consensus guidelines for pelvic exenteration focused on colorectal and gynaecological malignancies [21, 22]; however, only Laporte et al. [21] briefly emphasised the importance of functional follow‐up, including psychosocial and educational support to restore QOL. Multiple validated instruments and survey techniques for functional follow‐up were reported in the 19 studies, with the three most common being EORTC QLC‐C30, SF‐36, and FACT‐C. These instruments were often used at baseline and one other time point, which limited examination of a trend. Only six studies used their instruments at multiple time points in the post‐operative period, and most scores returned to baseline values at 1 year post‐operatively [52, 53, 54, 55, 57, 58]. Beyond QOL, other functional follow‐up domains were evaluated using tailored instruments for sexual function, faecal incontinence, or urogenital distress, or through symptom interviews conducted at routine clinic appointments. As a result, there is considerable variation in modalities and frequency of functional follow‐up within this patient population. Furthermore, while some instruments (e.g., EORTIC QLC‐C30 and FACT‐C) address global functional outcomes, no single instrument comprehensively assesses bowel, urinary, sexual, and lower limb function, along with pain.

### 4.1 Strengths and Limitations

To our knowledge, this systematic review is the first study to assess oncological and functional follow‐up strategies after pelvic exenteration surgery. As evidenced in the consensus guidelines for pelvic exenteration [21, 22], there are currently no follow‐up protocols specifically tailored for this patient population. Therefore, this study provides a comprehensive review of the follow‐up regimens adopted by various institutions worldwide. Although this review provides novel contribution, it is not without limitations. Notable limitations include the reliance on cohort studies (with no inclusion of RCTs), the potential for ‘higher’ risk of bias in some cohort studies, and the exclusion of scientific abstracts, which may have introduced selection bias and excluded institutions that did not formally publish their follow‐up protocols. Additionally, this study spans a 24‐year period during which advances in surgical techniques, multi‐disciplinary management, and evolving practice towards more complex pelvic exenterations, may influence long‐term follow‐up. These temporal changes, along with heterogeneity in tumour pathology (e.g., colorectal, gynaecological and urological), likely contribute to variability in follow‐up practice—including differences in imaging, surveillance frequency, laboratory testing, and functional assessments—and limits the interpretability of follow‐up outcomes across studies. Nevertheless, the strengths of this study include its adherence to standardised reporting frameworks, its use of robust risk of bias assessments, a comprehensive search strategy, and a broad scope addressing various malignancies that utilise the same surgical technique of pelvic exenteration.

## 5 Conclusion

This systematic review demonstrates the absence of standardised, procedure‐specific follow‐up protocols after pelvic exenteration and wide variation in surveillance intensity, modality and functional assessment. While some institutions extrapolate follow‐up schedules designed for primary colorectal cancer patients not requiring extended radical resection, this practice may have unknown implications for recurrence detection, function, and QOL in this high‐risk population. Robust multi‐centre observational studies, and ultimately, carefully designed RCTs are needed to develop practical, evidence‐based follow‐up protocols that are clinically meaningful, patient‐centred, and economically viable.

## Ethics Statement

The authors have nothing to report.

## Conflicts of Interest

The authors declare no conflicts of interest.

## Supporting information


**APPENDIX S1:** Search terms.

## Data Availability

The data that support the findings of this study are available from the corresponding author upon reasonable request.
