# Supplementary material for: Surveillance After Pelvic Exenteration: A Systematic Review of Oncological and Functional Follow‐Up
Source: ANZ J Surg. 2026 Feb 23;96(6):1481–97. doi: 10.1111/ans.70542 (PMC13327584; doi:10.1111/ans.70542)
Supplement: Supplementary file 1 — APPENDIX S1: Search terms. [file ANS-96-1481-s001.docx]

**Appendix 1. Search Terms**

**Question –** What constitutes appropriate follow-up after pelvic exenteration for locally advanced rectal cancer or locally recurrent rectal cancer? Clinician and patient perspective.

**Database:**

PubMed, Embase, Medline, and Cochrane library

**PubMed search:**

(“Pelvic exenteration”[MESH] OR “Pelvic exenteration”[Title/Abstract])

AND

("follow up"[Text Word] OR "long-term follow-up"[Text Word] OR "recurrence assessment"[Text Word] OR "recurrence evaluation"[Text Word] OR "Cancer follow-up"[Text Word] OR "followed up"[Text Word] OR "surveillance"[Text Word])

Filters: Humans, English

**Embase and Medline via OvidSP:**

(“Pelvic exenteration*”ti.kw.)

AND

("follow up" or "long-term follow-up" or "oncological follow-up" or "recurrence assessment" or "recurrence evaluation" or "Cancer follow-up" or "followed up" or "surveillance").tw.

Limit to English language

Limit to Human

**Cochrane search:**

(Pelvic exenteration) title abstract keyword

AND

("follow up" OR "long-term follow-up" OR "recurrence assessment" OR "recurrence evaluation" OR "Cancer follow-up" OR "followed up" OR "surveillance") all text
